# Making “Sense” of Ecology from a Genetic Perspective: *Caenorhabditis elegans*, Microbes and Behavior

**DOI:** 10.3390/metabo12111084

**Published:** 2022-11-09

**Authors:** Kyoung-hye Yoon, Rocel Amor Indong, Jin I. Lee

**Affiliations:** 1 Department of Physiology, Mitohormesis Research Center, Yonsei University Wonju College of Medicine, Wonju 26426, Korea; 2 Division of Biological Science and Technology, College of Science and Technology, Yonsei University, Wonju 26493, Korea

**Keywords:** nematode, bacteria, fungi, metabolites, sensory

## Abstract

Our knowledge of animal and behavior in the natural ecology is based on over a century’s worth of valuable field studies. In this post-genome era, however, we recognize that genes are the underpinning of ecological interactions between two organisms. Understanding how genes contribute to animal ecology, which is essentially the intersection of two genomes, is a tremendous challenge. The bacterivorous nematode *Caenorhabditis elegans*, one of the most well-known genetic animal model experimental systems, experiences a complex microbial world in its natural habitat, providing us with a window into the interplay of genes and molecules that result in an animal–microbial ecology. In this review, we will discuss *C. elegans* natural ecology, how the worm uses its sensory system to detect the microbes and metabolites that it encounters, and then discuss some of the fascinating ecological dances, including behaviors, that have evolved between the nematode and the microbes in its environment.

## 1. Introduction

Twenty years ago, the sequencing of the human genome was completed, ushering in a new genomic era that opened our understanding of how genes control the biology of an organism, from the most basic cellular functions to the physiology and behavior of an animal [1]. Molecular and genetic tools developed over the last few decades have revealed to us the contribution that a single gene can have on the development and life traits of an individual plant or animal. Today, with this plethora of genetic information, we can expand past single individual organisms and begin to ask even more challenging questions.

In particular, we can ask how organisms interact in nature at the genetic level and begin to answer more complex questions of animal ecology. For instance, animals can sense and interact with other organisms in their natural environments. These initial interactions within an animal’s community can lead to more complex competitive, predatory, or symbiotic behavioral relationships. Most of the known ecological relationships between organisms have been observed and studied in the field. However, with the onset of the genome era and new tools to study cells and animals molecularly and genetically, we can now study these ecological relationships at a finer resolution than ever before and begin to define how genomes interact at the ecological level. In particular, we want to know how animals can detect other organisms in their communities, how organisms communicate with one another, and how genes play a role in establishing ecological relationships between organisms. Such comprehensive analyses of animal ecology will paint a clearer picture of how ecological relationships may have evolved.

## 2. *C. elegans*: A Model for Genetic Studies and Ecological Studies in the Laboratory

Still, the ability to ask these deeper questions of ecology using molecular and genetic techniques is hindered by an inability to observe the ecology of animals in the laboratory. Although some laboratory studies have elucidated genes that may be involved in animal ecology, such as predator–prey relationships [2,3], the true ecology of most animal genetic models, including the fruit fly and the mouse, are too difficult to replicate within the laboratory environment. On the other hand, the free-living nematode *C. elegans* has been used as a genetic model animal for over 50 years in the laboratory. First identified in the 19th century [4] and studied in the laboratory in the 1950s [5], *C. elegans* has life trait qualities that make it amenable for laboratory genetic studies. The worm, as it is often called, can be cultivated on an agar plate seeded with bacteria, usually the common bacteria *Escherichia coli*. The hermaphrodite nematodes have a short three-day life cycle from hatching to sexual maturity, and lay 200–300 eggs over their lifetimes, with most of those eggs laid within the first few days of adulthood [6]. Since the hermaphrodites produce both sperm and eggs, their main mode of reproduction is by self-fertilization. However, males occasionally appear, allowing for cross-fertilizing sexual reproduction. Finally, *C. elegans* displays multiple visible phenotypes and behaviors that can be easily tracked. These characteristics of *C. elegans* led Sydney Brenner, in the 1960s, to choose the nematode as a genetic model to identify genes important for development and behavior [7,8]. Since then, the worm has been one of the most studied genetic model animals, resulting in seminal research that has led to multiple Nobel prizes.

*C. elegans* is one of the most studied animals in history; a search of the literature will reveal hundreds of thousands of published scientific papers. However, little is known about the natural ecology of *C. elegans*. Clues about how *C. elegans* may live and thrive in nature were inferred during laboratory culture. When cultivated on agar plates, the bacterivorous nematode quickly consumes all the bacterial food; if only a few adult hermaphrodites are placed on a seeded agar plate, all of the bacteria will be consumed within a few days, even before the 3rd generation reaches sexual maturity. However, when juvenile *C. elegans* larvae encounter stressful conditions, such as starvation, crowding or high temperature, they can enter an alternative developmental stage called dauer that will allow them to withstand harsh environments for longer periods of time [9].

These characteristics of *C. elegans* and other Rhabditidae nematodes are typical of colonizer or enrichment-type nematodes [10]. Unlike other types of nematodes that tend to persist in soil environments for long periods, colonizer nematodes take advantage of environments in which organism mortality or turnover has recently occurred, resulting in a sudden flush of microbial activity [11,12]. They will quickly grow, populating the environment in just days until the bacterial food is drained or crowded conditions move the larvae into the dauer stage [9,13].

Consistent with this, *C. elegans* can be found in nature seasonally in various soil environments with decaying fruits and vegetation, including apple orchards, vegetative composts, and forests [13,14,15,16], but almost never found in rotting leaves, grass or wood, nor plain soil [15,17]. In addition, *C. elegans* can be found in the gut of living slugs [18]. Each one of these microbe-rich environments provides the bacterivorous predator with essential prey that will allow development and rapid population growth. However, such abundant diversity of microorganisms in these environments can also expose *C. elegans* to microbial predators that look to prey on nematodes. Here, we look to review the literature and identify microorganisms that *C. elegans* may encounter and the molecules produced by these microbes that worms are exposed to. We will then discuss how *C. elegans* is able to detect or avoid these microorganisms, as well as how microbes can detect nematodes in their environments. Finally, we will describe the ecological relationships between *C. elegans* and microbes in their environment, as well as the behaviors that the worm performs in response to these microbes.

## 3. *C. elegans* Natural Interaction with Microbes

Microorganisms are essential for *C. elegans* biology, since the free-living hermaphroditic nematode thrives on substrates consisting of microbe-rich decomposing plant material. However, throughout much of the time of *C. elegans* research in the laboratory, microbial interaction was mostly limited to the OP50 *E. coli* strain, the standard laboratory diet for *C. elegans*. Only in the last decade or so, as thriving populations of *C. elegans* have been found in rotting fruits and plants, the ecological relationship with microbes began to be explored. *C. elegans*’ association with bacteria can be roughly divided into three categories: food, gut microbe or pathogen. On the other hand, fungi mostly play the role of either pathogen or predator [17,19,20]. *C. elegans* is also found in nature infected with *Microsporidia* and viruses [21,22], but in this review, we will focus on bacteria and fungal species.

### 3.1. Bacteria

Several studies have investigated the bacterial communities in the natural habitat of *C. elegans*. Samuel et al. sequenced the microbes inside rotting apples and found a community of Proteobacteria, Bacteroidetes, Firmicutes and Actinobacteria. Interestingly, differences in bacterial composition and relative abundance could be observed in apples where *C. elegans* was found versus those where none were found. In apples where a proliferating population of *C. elegans* was found, an enrichment for *Acetobacteriaceae* was observed, and less of *Chryseobacterium*, *Flavobacterium*, *Pseudomonas*, *Sphingomonas*, *Stenotrophomonas* and *Xanthomonas* were found. Overall, *C. elegans* tended to be found in apples with a simpler microbial composition [13].

Worms in the wild were often found with bacteria in the gut. Sequencing these microbes revealed that *Proteobacteria*, especially *Enterobacteriaceae*, and the genera *Pseudomonas*, *Stenotrophomonas*, *Ochrobactrum* (which persisted in the gut even after switching to OP50) and *Sphingomonas* were the dominant taxonomic groups [21,23]. Of note is that the bacteria in the gut was found to be distinct from the microbial composition of the surrounding environment, and different even from a closely-related species isolated from the same place, indicating that there is a selective process that determines which ones stay in the gut [23,24,25]. Many of the bacteria that made up the gut microbes was shown to have a positive effect on the worm, such as improved growth and fecundity, or increased immunity and resistance to pathogens [13,23,26,27].

While most bacteria support worm growth, plenty are pathogenic. *C. elegans* are susceptible to a wide range of pathogenic bacteria [28]. Pathogen infection and immunity in *C. elegans* has been extensively studied, and it was found that the worms mount an immune response that is both general and specific for the pathogen and site of infection [29,30].

In the natural setting of rotting apples, Samuel et al. found that ~20% of the bacteria isolated in the rotting fruit environment was detrimental or pathogenic [13]. For some, the detrimental effect could be mitigated by mixing with beneficial bacteria, suggesting that some beneficial bacteria can actively provide antivirulence effects. This is an example of the multi-genome interaction which is likely common in nature. Studies using combinations of mutant bacteria and mutant *C. elegans* will further reveal the intricacies of the genomic interaction between bacteria and nematodes [31,32].

### 3.2. Fungi

Studies on fungal infection in *C. elegans* have yielded insights into the detailed mechanism of fungal pathogenesis and antifungal immunity in the nematode. The nematode-specific fungus *Drechmeria coniospora* has long been used to study anti-fungal immunity in *C. elegans*, which revealed a set of innate immune responses distinct from those of bacterial infection [29]. *D. coniospora* attaches to the cuticle at the mouth or vulva and invades the nematode by the hyphae, colonizing the worm [33]. Other fungal species that more actively “hunt” for nematodes include the nematode-trapping fungi (NTF), such as *Arthrobotrys oligospora* and *Duddingtonia flagrans,* which develop specialized trap structures to capture worms [19,34,35]. In addition, there are also fungi that use toxins to quickly immobilize and kill the nematodes, such as *Pleurotus ostreatus* [20]. The various highly specialized strategies for targeting nematodes evolved independently across many fungal lineages, providing fascinating examples of interspecies interaction and the evolutionary arms race [36,37].

## 4. Microbial Metabolites and Their Effect on *C. elegans*

Bacteria produce and secrete abundant proteins, chemicals and gases into the surrounding environment. Most of these secondary metabolites, metabolites that are produced and secreted during the stationary phase of growth, function to benefit the growth of bacteria and bacterial communities [38]. Autoinducers, such as homoserine lactones, are involved in quorum sensing to allow crosstalk between bacterial communities [39]. Other secondary metabolites can be used as weapons against bacterial competitors, such as antibiotics, or predators, such as bacterivorous nematodes [40].

Many of the secreted secondary metabolites have biological effects on *C. elegans*. For instance, many of the metabolites are toxic to *C. elegans* [41,42,43,44]. Violacein, a bis-indole secondary metabolite produced in a diverse array of bacteria [45], was found to be toxic to *C. elegans* adults, and to arrest *C. elegans* larvae growth and development [41,46]. A number of metabolites have been reported to improve nematode health and increase lifespan [44,47,48,49,50] and even prevent neurodegeneration [51]. For instance, nitric oxide gas produced by bacteria can increase *C. elegans* lifespan [50], and the *E. coli* metabolites GABA and lactate can have neuroprotective effects against progressive touch sensory neuron degeneration in worms [51]. Metabolites can also elicit *C. elegans* attraction and repulsion behavior [34,52,53,54,55,56]. Two secondary metabolites, produced by *Pseudomonas aeruginosa*, phenazine-1-carboxamide (PCN) and pyochelin, were reported to promote avoidance behavior in *C. elegans*. These metabolites were observed to activate a G protein-signaling pathway in the ASJ chemosensory neuron pair, which induces expression of the neuromodulator DAF-7/TGF-ß in *C. elegans*. DAF-7 activates a TGF-ß signaling pathway in adjacent interneurons to modulate aerotaxis behavior and promote avoidance [52].

## 5. Detection of Metabolites by *C. elegans* Sensory System

Sensory detection of most microbial metabolites occurs largely through chemosensory G protein-coupled receptors (GPCRs) expressed in the sensory neurons. Most animals devote a large portion of their genome to a diverse repertoire of chemosensory receptors in order to ensure the detection of varied cues they come across in nature, and this is no exception for *C. elegans*. *C. elegans* is estimated to have ~1300 chemosensory GPCRs, which represents approximately ~5% of the genome [57]. This is quite a large number, especially considering its simple nervous system and limited number of sensory neurons. Each sensory neuron expresses multiple chemosensory receptors, which is different from how most animals regulate chemosensory receptor in the nose, where only one receptor is expressed in each neuron to ensure discrimination of chemical cues [58]. This presents an outstanding question in the field, of how discrimination is achieved in such an arrangement, or whether there is discrimination at all between chemicals detected by the same set of neurons. One method for discrimination between chemicals is through beta-arrestin-mediated desensitization of previously activated receptors, so that neurons can respond to new chemical cues [59].

Genomic analysis has revealed that, even within nematodes, there is much variability in the numbers of chemosensory receptors. *C. briggsae* is reported to have 452 [60], although this seems to be an undercount due to unannotated GPCR genes in the *C. briggsae* genome (PFAM ver35). The closest cousin to *C. elegans*, *C. inopinata*, has less than 400. The much smaller number is thought to reflect the type of ecological niche of the nematode: whereas *C. elegans* need to detect a wider variety of chemical cues living in decaying vegetation whose condition constantly fluctuates, *C. inopinata* mainly reside in figs. This very specific and, thus, consistent environment precludes the need for diverse receptors. Such is the case in the parasitic nematodes as well, which stay in a consistent environment inside the host [61]. This also reflects the fast-evolving nature of the chemosensory receptor gene family, contracting and expanding through pseudogenization and gene duplication, to fit the needs of the organism.

To date, only a few receptor–ligands pairs have been identified among *C. elegans* chemosensory receptors. Contrary to neuropeptide GPCRs [62], successful chemosensory GPCRs expression in mammalian cell lines have been rare, although a few have been successfully expressed and deorphanized [63,64]. Some were identified by testing mutants of chemosensory GPCRs that do not respond to the chemical cue [65,66,67,68,69,70]. However, due to publicly available single-cell RNAseq data, such as CeNGEN [71], and highly optimized protocols for the CRISPR-Cas9 genome editing technique, we may see more deorphanization in the near future. Important to note is that each chemical cue is likely detected through several receptors activating at once, each with varying affinity, much like the combinatorial coding of odor receptors in mammals [72]. This redundant detection could present another challenge to identifying ligand–receptor pairs, as in some cases, deleting one receptor may produce only a subtle change in behavior or neuron activation.

## 6. Microbial Metabolites Elicit Innate but Plastic Behaviors

*C. elegans* respond to many metabolites by either attraction or aversion. These responses are hardwired by neurons that respond to the cues, which suggests there is an ecological significance to these metabolites. Odors that have been traditionally used to study *C. elegans* olfaction are volatile metabolites produced by various microorganisms. One such odor, diacetyl, is attractive to *C. elegans* and is produced in yeast or lactic acid bacteria, whose biosynthesis process has been well-studied for industrial purposes [73]. Interestingly, diacetyl is produced by *Lactobacillus* only when citrate is available. Therefore, diacetyl may not only signal the presence of lactic acid bacteria, but it may also serve as a signal to the presence of a microbial community that is supported by a citrate-rich environment, like rotting citrus fruit. Consistent with this, a strain of *Lactobacillus paracasei* and *Caenorhabditis remanei* has been isolated in rotten yuzu, a citrus fruit native to East Asia, demonstrating that the two can be found cohabiting the same environment [73].

The innate preference for many volatile metabolites can change depending on the context. The main receptor that detects diacetyl is ODR-10, which is one of the few deorphanized receptors in *C. elegans* [65]. Interestingly, expression of ODR-10 is modulated in a sex-, developmental stage- and life history-dependent manner [74,75]. For example, compared to hermaphrodites, adult male *C. elegans* are much less attracted to food odors such as diacetyl, to prioritize mating. However, if the male worms are starved, they increase ODR-10 expression, resulting in increased attraction to diacetyl and, thus, food. Similarly, post-dauer worms, having experienced starvation, showed increased expression of ODR-10 and attraction to diacetyl. In this case, attraction was not limited to diacetyl, but to a wide range of food-related odors, consistent with the increased chemosensory receptor expression observed in post-dauer worms [75]. Such context-dependent regulation of receptor expression allows the worms to appropriately respond to the microbial environment, resulting in increased fitness. The attraction of *C. elegans* to diacetyl was also shown to diminish in the absence of the germline, specifically in hermaphrodite adults, demonstrating that odor behavior is also modulated by reproductive status [76]. However, decreased ODR-10 levels were not observed, suggesting the diminished response occurs through a different mechanism.

Another context to consider is the concentration of the particular cue. It is well established that many attractive odors are repulsive at high concentrations [77]. In the case of the bacterial metabolite dimethyl trisulfide (DMTS), attraction and repulsion are mediated by the same receptor expressed in different neurons. Low concentration of DMTS activates SRI-14 in the attraction-mediating AWC sensory neuron, and high concentration activates SRI-14 in the repulsion-mediating ASH sensory neuron in order to generate the appropriate response [64].

Volatile chemical cues are also used for *C. elegans* to avoid pathogenic bacteria. Some pathogenic bacteria, such as *Serratia marcescens*, emit odors that initially make it more attractive to *C. elegans* than nutritive bacteria [54,78]. However, once they graze on the bacteria, they learn to avoid them in the future [79]. This is accomplished by associating the bacteria with its various metabolites: in the case of *P. aeruginosa* (PA14), the non-volatile pyochelin and phenazine-1-carboxamide, as well as volatile odorants, mediate the learned avoidance [52,79]. Interestingly, pathogen odor not only elicits innate or learned behavior, but can also act as a signal to prepare for the imminent infection. A study by Ooi and Prahlad reported that just being exposed to the PA14 odor primes a naive worm to mount a faster stress response and avoidance behavior once they encounter the pathogen [80]. They showed that pre-exposure to PA14 odors such as 2-aminoacetophenone caused the heat shock transcription factor HSF-1 to form punctae in the nucleus, presumably binding to the regulatory element of downstream target genes. This allows for quicker transcription upon exposure to the pathogen. In addition, PA14-derived odor 1-undecene can activate immunity [81]. These studies show that volatile metabolites are ecologically relevant cues, whose sensation through the sensory neurons elicit not only behavior, but relevant physiological response in other parts of the body.

## 7. Interspecies Interaction through Gas Sensing

Aside from volatile chemical cues, bacteria also emit gas, namely carbon dioxide (CO_2_) and nitric oxide (NO), as byproducts of cellular respiration, and consume oxygen (O_2_), whose ambient concentration can all be detected by *C. elegans*. Presence of these gases in different concentrations can signal the presence of food, potential predators, or in the case of dauers, phoretic opportunities. Correspondingly, as is the case for volatile metabolites, worms show attraction or repulsion towards different concentrations of gases, but the response is highly context-specific and varies depending on the developmental stage, past experience, and nutritional status. For example, N2 adults strongly avoid CO_2_, whereas dauers are strongly attracted to it [82]. This negative valence to CO_2_ in adults can be switched in a matter of a few hours when either starved or cultivated in high-CO_2_ environments, becoming neutral or even attractive [83,84,85]. In addition, aerotaxis experiments show that concurrent stimuli, such as presence of food, shifts the preferred O_2_ concentration range of *C. elegans* [86].

Gases are sensed by atypical soluble guanylyl cyclases. Increasing concentration of O_2_ is sensed by GCY-35 and GCY-36 expressed in the AQR, PQR and URX neurons, and decreasing concentration is detected by GCY-31 and GCY-33 in BAG. CO_2_ is sensed by GCY-9 expressed in the BAG sensory neurons [82,87]. ASE and AFD, usually associated with salt and heat sensation, respectively, can also respond to CO_2_ [84]. NO is sensed by guanylyl cyclase DAF-11 in the ASJ neuron [88]. The oxygen sensing GCY-35 has been also shown to bind to NO, at least in vitro [89].

Most wild strains of *C. elegans* prefer O_2_ concentration between 5–12%, with the highest preference at 10% [86]. This corresponds to their natural habitat of decaying vegetation. However, N2, having long been cultured in laboratory conditions of bacterial lawn grown on top of agar media, has accumulated mutations that cause it to prefer or tolerate higher O_2_ concentrations. The most prominent is the gain-of-function mutation in the neuropeptide Y receptor homolog npr-1 allele in the N2 strain, that results in lower neuronal activity in the AQR, PQR and URX O_2_-sensing neurons [90,91,92]. N2 strains also have a loss of function mutation in the neuroglobin gene glb-5, a heme-binding protein that modulates the sensitivity of the URX neuron to small changes in O_2_ [93,94].

NPR-1 activity also affects the behavioral response to CO_2_. While N2 is repelled by CO_2_, the wild Hawaiian strain shows a neutral response, despite the same levels of BAG neuron activity [95]. This is due to the low activity of NPR-1 in the Hawaiian strain, resulting in higher activity in the URX neurons, which in turn inhibits the avoidance behavior, likely through the release of FLP-8 and FLP-19 neuropeptides [95].

Aside from attraction and avoidance, O_2_ and CO_2_ sensation are also inextricably linked to foraging behavior, such as aggregation and bordering on bacterial lawns, on-food speed and food leaving. Using the same neurons, the animal integrates the surrounding gas concentrations with internal state or concurrent cues, such as temperature or pheromones, to modulate these behaviors [89,90,96,97,98,99,100,101].

*C. elegans* lacks nitrogen oxide synthase (NOS) to synthesize NO, but can detect NO in the environment. NO exposure produces physiological responses in the worm, such as stress response induction and increased lifespan [50], but it can also be detected through the ASJ sensory neurons, eliciting an avoidance response [88].

## 8. Ecological Relationships: *C. elegans* and Bacteria

Bacteria are vulnerable to predators such as bacterivorous nematodes, which are abundant in all types of natural ecosystems where microbes are present. In order to defend themselves, bacteria use strategies such as the formation of biofilm communities. Biofilms are multicellular communities of bacteria surrounded by an expolymer matrix [102]. However, mechanisms of how biofilms may protect bacteria from *C. elegans* predation has only recently been revealed.

Formation of a biofilm requires crosstalk between individual cells. During biofilm formation, bacterial cells secrete quorum-sensing (QS) autoinducing compounds, such as homoserine lactones, which induce other cells to form a biofilm [39]. However, other bacterivorous organisms can become illegitimate receivers of those signals and also detect these autoinducers. *C. elegans* can detect N-butanolyl homoserine lactones (BHLs) produced by *P. aeruginosa* during biofilm formation using the diacetyl receptor ODR-10, and are attracted to BHLs that can allow the worms to find the bacteria. Mutants of *odr-10* cannot detect BHLs [103]. One strategy to prevent *C. elegans* from easily finding *P. aeruginosa* by olfaction is to “cloak” the QS autoinducing chemicals with the biofilm matrix itself. The biofilm matrix is composed of an expolysaccharide released by the bacteria, and the expolysaccharides themselves prevent QS autoinducers from leaking out into the surrounding environment. Mutant *P. aeruginosa* that lack components of the expolysaccaharide matrix are unable to keep the QS autoinducers within the biofilm, and the leaked BHLs can then be sensed by *C. elegans,* which find and consume the bacteria [103].

Masking odors is not the only way biofilms can prevent *C. elegans* predation of *Pseudomonas* bacteria. Even if worms reach the biofilm matrix, the matrix itself impedes *C. elegans* motility. Bacterivorous nematodes like *C. elegans* use their movement to effectively graze on bacteria in their environments. However, worms significantly slow their movement on the normal biofilm matrix, whereas on the biofilms of mutant *P. aeruginosa* strains that lack certain expolysaccharides, *C. elegans* movement is restored [104]. It is thought that the rigid polymer may hinder normal movement of *C. elegans,* restricting its ability to graze on the bacteria, allowing more bacteria to survive predation.

Of the many secondary metabolites produced by bacteria during quorum sensing, the purple pigmented compound violacein is produced by diverse types of bacteria, including *Janthinobacterium*, *Duganella* and *Chromobacterium* [45]. Violacein is toxic to *C. elegans*, resulting in intestinal distension and eventual shortened lifespan of adult worms [41,46]. Moreover, violacein is particularly toxic to the young larvae, arresting development at the 1st larval stage and eventually killing all of the larvae [46]. *C. elegans* mothers, however, have evolved a fascinating way to allow some of their young to survive the toxicity of the bacterial metabolite. Upon exposure to violacein, a *C. elegans* hermaphrodite will cease laying eggs and allow the eggs inside her body to hatch within her [46]. This behavior, called matriphagy, allows the young to consume the mother from the inside out, eventually eating their way out of their mother’s carcass. Although the young larvae will then be exposed to violacein, some will now be able to escape the toxicity and developmental arrest and proceed to adulthood. The researchers found that consuming their mother’s body provides the young with a nutrient, oleic acid, that allows the young to survive the toxicity of the secondary metabolite.

## 9. Ecological Relationships: *C. elegans* and Fungi

In enriched soil and rotting fruit environments, *C. elegans* commonly encounter diverse types of fungi. However, some species of fungi, aptly termed nematode-trapping fungi (NTF), have turned the tables on the bacterivorous predator. These filamentous fungi, which include over 700 different species [105], live in soils as saprotrophs, but in low nutrition conditions and in the presence of nematodes, they will switch to a predatory feeding mode [105,106]. NTFs use adhesive networks and other various structures to trap nematodes, immobilizing them and finally penetrating and digesting the nematodes with various enzymes [105,106,107]. NTFs are so effective at killing nematodes that they are used in agricultural applications as a remedy for parasitic nematodes [108,109].

Although NTFs are common and diverse, the mechanism of nematode trapping is often species-specific, involving a rather complex interplay between secreted compounds from both fungi and nematodes. For instance, the fungus *Duddingtonia flagrans* produces three-dimensional adhesive trap networks that can trap and immobilize nematodes [106]. However, these traps are formed only in the presence of nematodes. Trap formation is controlled and inhibited by the compound 6-MSA and its derivative, arthrosporols, that are synthesized in the fungal hyphal tips [34]. Interestingly, *C. elegans* can sense and is attracted to 6-MSA, allowing the nematodes to approach the nematode-trapping fungi. However, *C. elegans* also produces ascarosides, chemical pheromones that allow the worms to communicate to each other. *D. flagrans*, as an illegitimate receiver of the pheromones, also receives the ascaroside signals [34]. The nematode ascarosides represses the biosynthesis of 6-MSA and arthrosporols, allowing the formation of the trap network. After nematodes are trapped, hyphae can penetrate the worm, exposing the worms to fungal secretions which, in *D. flagrans,* consist of more than 200 specific small-secreted proteins (SSP) that have no similarity to any known proteins or enzymes [110]. Among these, *CyrA* is an SSP that is secreted from bulbous structures formed after the hyphae have entered the *C. elegans* body [111]. *CyrA* increases the toxicity of *D. flagrans*, as the *CyrA* mutant fungus was less virulent than the normal strain. Although its mechanism of toxicity is unknown, *CyrA* can be found in the worm coelomocyte cells, which are scavenger cells thought to inactivate the small protein [111].

Microbes that feed on *C. elegans* use volatile cues to their advantage and emit odors (volatile metabolites) to lure their prey. *Arthrobotrys oligospora*, a fungi that feeds on nematodes to supplement their nitrogen intake, shows full utilization of nematodal chemical cues to their advantage: in addition to emitting an odor that mimics a nematode pheromone to attract and trap them, *A. oligosopora* is an illegitimate receiver, eavesdropping on nematode pheromones to detect their presence. This allows them to conserve energy, forming traps only when potential prey is around [35,112].

Another predatory fungi that has been studied in detail is the oyster mushroom *Pleurotus ostreatus*. Instead of trapping the nematodes, *P. ostreatus* paralyzes and kills its prey. Whereas NTFs penetrate the cuticle with their hyphae, the *P. ostreatus* toxin enters the worms through the sensory neuron cilia in the amphid pore, located in the head of the worm. Mutant worms with defects in cilia formation are resistant to toxin entry. The toxin is effective for diverse nematode species, exposing a vulnerability in most nematodes that may be exploited to develop novel anthelmintics [20].

## 10. Conclusions and Future Perspectives

Genetic experiments over the last few decades have revealed the functions of specific genes in many organisms, including *C. elegans* and many types of microbes. These experiments, as we have discussed, have elucidated how *C. elegans* senses its environment and how bacteria communicate with one another and begin to form biofilms. However, how these genes, structures and even communities interact with one another—forming ecological relationships in nature—has been more difficult to ascertain. The rather simple ecosystem in which *C. elegans* and microbes inhabit provides us a window to understand such complex ecology. Moreover, simulating the *C. elegans* habitat in the laboratory in the future would allow us to better understand ecological relationships, but also help us to better understand the functions of genes themselves. By challenging worms in their natural environments, we may be able to identify new functions or hidden functions of genes, and give further clues to how genomes may have evolved.
